# Association of cesarean section with asthma in children/adolescents: a systematic review and meta-analysis based on cohort studies

**DOI:** 10.1186/s12887-023-04396-1

**Published:** 2023-11-16

**Authors:** Ziwei Zhong, Meiling Chen, Senjie Dai, Yu Wang, Jie Yao, Haojie Shentu, Jianing Huang, Chiyuan Yu, Hongrui Zhang, Tianyue Wang, Wei Ren

**Affiliations:** 1 Emergency Medical Center, Ningbo Yinzhou No. 2 Hospital, Ningbo, Zhejiang China; 2 The Public Health College, Zhejiang Chinese Medical University, Hangzhou, Zhejiang China; 3 The Second Clinical Medical College, Zhejiang Chinese Medical University, Hangzhou, Zhejiang China; 4 College of Pharmacy, Hangzhou Medical College, Hangzhou, Zhejiang China; 5 The Medical Imaging College, Hangzhou Medical College, Hangzhou, Zhejiang China; 6 The Medical Technology and Information Engineering College, Zhejiang Chinese Medical University, Hangzhou, Zhejiang China; 7 General Family Medicine, Ningbo Yinzhou No. 2 Hospital, 998 North Qianhe Road, Yinzhou District, Ningbo, 315100 Zhejiang China

**Keywords:** Cesarean section, Asthma, Child, Adolescent, Offspring, Meta-analysis

## Abstract

**Background:**

Whether cesarean section (CS) is a risk factor for asthma in offspring is controversial. The purpose of this study was to investigate the association between CS and asthma in children/adolescents.

**Methods:**

Pubmed, Embase, Web of Science, and Cochrane Library electronic databases were searched for cohort studies on the relationship between mode of delivery and asthma in children/adolescents up to February 2023. Birth via CS was considered an exposure factor. Asthma incidence was taken as a result.

**Results:**

Thirty-five cohort studies (thirteen prospective and twenty-two retrospective cohort studies) were included. The results showed that the incidence of asthma was higher in CS offspring (odds ratio (OR) = 1.18, *P* < 0.001) than in the vaginal delivery (VD) group. Partial subgroup analyses showed a higher incidence of asthma in female offspring born via CS (OR = 1.26, *P* < 0.001) compared with the VD group, while there was no difference in males (OR = 1.07, *P* = 0.325). Asthma incidence was higher in CS offspring than in the VD group in Europe (OR = 1.20, *P* < 0.001), North America (OR = 1.15, *P* < 0.001), and Oceania (OR = 1.06, *P* = 0.008). This trend was not found in the Asian population (OR = 1.17, *P* = 0.102). The incidence of atopic asthma was higher in offspring born via CS (OR = 1.14, *P* < 0.001) compared to the VD group. The CS group had a higher incidence of persistent asthma, but the difference did not reach statistical significance (OR = 1.15, *P* = 0.063).

**Conclusion:**

In this meta-analysis, CS may be a risk factor for asthma in offspring children/adolescents compared with VD. The relationship between CS and asthma was influenced by sex and region.

**Supplementary Information:**

The online version contains supplementary material available at 10.1186/s12887-023-04396-1.

## Introduction

Asthma is one of the leading causes of chronic respiratory disease-related death globally [1] and is the most common noncommunicable disease. Globally, asthma affects about 300 million people [2], and the prevalence of asthma in children and adolescents is approximately 10% [3]. Patients often suffer recurrent episodes of wheezing, coughing, chest tightness and other symptoms [4, 5]. Chronic airway inflammation is a common feature of asthma. It not only causes adverse physical and psychological feelings, but also lowers the quality of life and shortens life expectancy. Asthma is caused by the interaction of genetic and environmental factors [6]. Lung function defects, respiratory infections, and other factors are associated with the development of asthma [7]. Fetal exposure during pregnancy (such as cesarean section (CS) [8]) has been suggested as one of the determinants of immune system development [9].

In recent decades, the incidence of CS worldwide has continued to rise. The global CS rate is expected to increase to nearly 30% in 2030 [10]. Although rational use of CS in critical settings can reduce maternal and neonatal mortality and morbidity [11], excessive use of CS is not beneficial to the mother or the infant and will cause some waste of resources [12–14]. Even if the application of CS is mature, the short or long-term health damage of CS to infants is worth exploring, such as obesity [12, 15], type 1 diabetes [16, 17], and leukemia [18]. In addition, CS has been considered to increase the risk of asthma in offspring [19, 20]. Asthma is related to genetic factors [6] and varies from country to country, necessitating a comprehensive analysis of asthma risk in different regions. So far, the relationship between CS and asthma has been controversial. Some meta-analyses have shown that CS increases the risk of asthma in children. However, there are limitations, such as the small number or the small regional scope of included studies [12, 21]. Another meta-analysis from the European region found that due to the heterogeneity of results, CS cannot be explicitly considered a risk factor for asthma [22]. Therefore, this is an updated systematic review and meta-analysis that intends to include cohort studies with higher ability to test causality. The aim was to explore the relationship between CS and asthma in children/adolescents by reviewing previous relevant studies.

## Method

### Literature search strategy

This systematic review and meta-analysis was conducted according to the Preferred Reporting Items for Systematic Reviews and Meta-Analyses (PRISMA) [23] and Meta-analysis of Observational Studies in Epidemiology guideline [24]. Two researchers systematically searched data from studies on the relationship between CS and asthma in Pubmed, Embase, Web of Science, and Cochrane Library electronic databases until February 2023. Retrieve based on the following keywords: "Cesarean Section" [Mesh] and "Asthma" [Mesh]. Please refer to Supplementary Table 1 for detailed search strategies. In addition to this, the reference list of relevant literature was manually reviewed to avoid missing studies. The systematic review and meta-analysis Prospero registration number is CRD42023420333.

### Inclusion and exclusion criteria

The following inclusion criteria were used in this systematic review and meta-analysis: (1) the article assessed the relationship between mode of delivery (CS vs. vaginal delivery (VD)) and asthma in offspring. (2) The exposed group was offspring born via CS and the control group was offspring born via VD. (3) The study was a cohort study.

Exclusion criteria were as follows: (1) the study was not published in English. (2) Relevant data could not be extracted. (3) The study was not conducted with children/adolescents (age > 18y). (4) Only the study protocol or ongoing study was available, or the full text was not available. When multiple updates of the same cohort study were published, the most comprehensive or recent article was included.

### Quality assessment and data extraction

The Newcastle–Ottawa Quality Assessment Scale (NOS) checklist was used to assess the included cohort studies. Two researchers used a pre-designed form to extract the following information: author, year, country, study design, number of study subjects, age at diagnosis of asthma, asthma registry, data source, and adjustment factors. Third-party researchers resolved disputes.

### Objectives and outcomes

The aim of this study was to assess the association between CS offspring and asthma incidence. The outcome was that participants were diagnosed with asthma during children/adolescences (age ≤ 18y). The pre-designed subgroups were CS type, offspring sex, and asthma type (atopic asthma, seasonal asthma, drug-induced asthma, pulmonary asthma, etc.).

### Statistical analysis

Data were analyzed in Stata software version 12.0 to integrate estimates extracted from the included studies. The relationship between CS and asthma incidence was assessed by odds ratio (OR) and 95% confidence interval (CI). The Cochran Q chi-square test and the I^2^ statistical test were used to quantitatively assess heterogeneity between studies. A two-sided *P* < 0.1 of the Q test or I^2^ > 50% was considered statistically significant heterogeneity. Considering factors such as different CS types and ethnic populations, a random-effects model was used to improve the confidence in the results. Begg’s test was used to assess publication bias. Sensitivity analyses were used to determine the impact of individual studies on the overall risk assessment. A two-sided *P* < 0.05 was considered statistically significant.

## Results

### Study selection

**Fig. 1.**
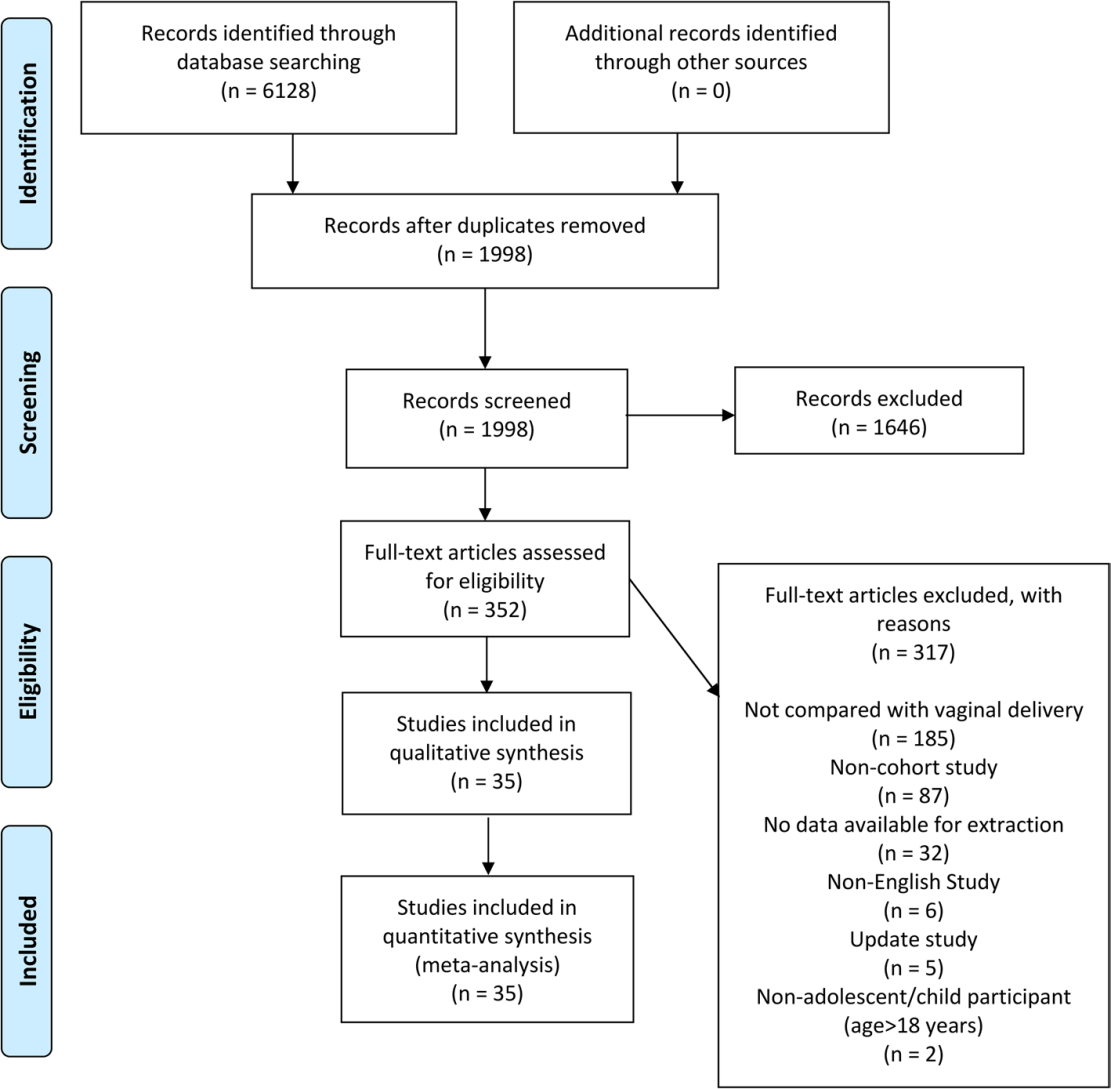
PRISMA retrieval procedures flow chart

In this study, 6,128 relevant articles were identified in four electronic databases, with 1,998 articles remaining after removing duplicates. There were 352 articles that met the inclusion criteria for titles and abstracts. We carefully and thoroughly reviewed the articles, of which 317 were excluded for various reasons, and 35 articles [19, 20, 25–57] were finally included in the systematic review and meta-analysis. A detailed PRISMA flowchart is shown in Fig. 1.

### Study characteristics and quality assessment

Table 1 shows the basic characteristics of all included articles. Thirteen prospective cohort studies and twenty-two retrospective cohort studies were included in the systematic review and meta-analysis of the association between CS and asthma. These selected studies were distributed across different regions, with four studies conducted in Asia, twenty-three studies in Europe, five studies in North America, and three studies in Oceania. Participants were diagnosed with asthma between the ages of 0–18. Most articles adjusted for confounding factors such as sex, gestational age, birth weight, maternal age, and parity. Supplementary Table 2 provides details about the source of the participants, birth period, follow-up time, and asthma registry. The quality of the included studies was assessed by the NOS checklist with a score of ≥ 6. Detailed results of the quality assessment are provided in Supplementary Table 3.

**Table 1:** Characteristics of all the studies included in the systematic review and meta-analysis

Author	Year	Country	Continent	No. of Paticipants		Diagnostic age (year)	Ajusted factors	
							Child factors	Maternal factors
				CS	VD			
Nafstad [25]	2000	Norway	Europe	279	2,193	4.3	NA	
Xu [26]	2000	Finland	Europe	1,147	6,386	7	Sex, GA, BW, birth seasons	Age, parity, pregnancy (smoking, BMI before, weight gain), allergic disorders history, status, social class
Annesi-Maesano [27]	2001	UK	Europe	NA	NA	1–18	Sex, age, BW < 2.5 kg, prematurity, birth order, sibship size	Age, parity, pregnancy smoking, asthma
McKeever [29]	2002	UK	Europe	4,073	18,573	0–11	NA	
Kero [28]	2002	Finland	Europe	8,826	51,039	7	Sex, BW	Age, previous deliveries
Maitra [30]	2004	UK	Europe	1,387	10,980	5–8	Sex, BW, preterm delivery, breastfeeding duration	Age, asthma/eczema and hayfever, pregnancy smoking, environmental tobacco smoke exposure at 6 months, education, number of 0–15 years old in household, crowding in home, damp housing, household cats, ethnicity
Bernsen [31]	2005	Netherlands	Europe	85	1,627	6	Sex, sibship size, birth order, birth season, birth year, age at the check-up time, diphtheria tetanus pertussis poliomyelitis vaccination status	Age, atopic disease (parents), occupation of the breadwinner level, urbanization level, origin country
Renz-Polster [33]	2005	USA	North America	1,286	6,586	3–10	Sex, BW, birth order, diagnosis age	Age, pregnancy smoking, multiple gestation, asthma/hay fever medications use, exposure to antibiotics in the postpartum period, marital status, education, ethnicity
Juhn [32]	2005	USA	North America	714	6,392	7	Sex, BW	Age, education
Salam [34]	2006	USA	North America	717	2,747	8–17	Sex, GA, BW, birth order, birth calendar period, requirement for special care after birth	Age at childbirth, asthma and allergy history (parents), pregnancy smoking, environmental tobacco smoke exposure, disease history (pneumonia, bronchitis, bronchiolitis, croup), parents or guardians education, health insurance coverage, residence community, race
Werner [35]	2007	Denmark	Europe	841	6,278	≤ 18	Sex, breastfeeding	Age, previous deliveries, pregnancy smoking, educational level
Pistiner [36]	2008	USA	North America	102	330	9	NA	
Tollånes [37]	2008	Norway	Europe	136,735	1,520,088	0–18	Sex, birth order, birth year	Age, asthma, education
Roduit [38]	2009	Netherlands	Europe	247	2,670	8	Sex, BW, breastfeeding	BMI, education, allergy status (parents)
Park [40]	2010	Korea	Asia	100	179	4.6 ± 3.8	Sex, GA, BW, breastfeeding	Age, allergy (parents)
Davidson [39]	2010	UK	Europe	18,462	230,150	2–11	Sex, GA, BW, breastfed or not, Apgar 1, birth year	Age, parity, smoking, asthma, forceps delivery, marital status, social class
Magnus [41]	2011	Norway	Europe	5,020	32,151	3	Sex, GA, BW, exclusive breastfeeding duration, childcare attendance	Age, parity, pregnancy (smoking, BMI before, chronic conditions before, complications), indication of personal preference for CS, previous delivery by CS, atopy, membrane rupture indication, marital status, educational level
Almqvist [42]	2012	Sweden	Europe	16,460	145,341	> 10	Sex, GA, BW, birth order, Apgar score, hypoxia/asphyxia	Age, pregnancy smoking, birth country, BMI, living with child father
Bråbäck [43]	2013	Sweden	Europe	29,925	143,347	2–5; 6–9	Sex, GA (small or large), birth year, meconium aspiration, neonatal respiratory distress, transient tachypnoea	Age, smoking, education, BMI, chorioamnionitis, history of diabetes and hypertension, premature rupture of the membranes, preeclampsia, pregnancy diabetes, HDP, fever during labour, asthma medication (parents), social welfare, urban/rural living, county
Pyrhönen [44]	2013	Finland	Europe	551	2,630	1–4	Sex, BW, birth order, breastfeeding duration	Pregnancy (smoking, duration), allergy (parents)
Black [45]	2015	UK	Europe	68,370	252,917	5	GA, BW, birth year, male infant, breastfeeding at 6 weeks	Age, smoking status, salbutamol prescription, Carstairs decile
Brüske [46]	2015	Germany	Europe	389	1,461	15	Sex, GA	Education level (parents), study center
Kristensen [48]	2016	Denmark	Europe	124,130	666,439	0–14	Sex, GA, BW	Age, pregnancy smoking, pregnancy complications (preeclampsia, eclampsia, hemorrhage, and hyperemesis)
Sevelsted [49]	2016	Denmark	Europe	163,462	696,864	0–15	Sex, GA, BW, calendar year	Age, multiple births, parity, pregnancy (smoking, antibiotics), employment status, asthma
Black [47]	2016	UK	Europe	26,766	13,379	> 6	GA, male infant, BW, birth year, breastfeeding at 6 weeks	Age, smoking status, salbutamol prescription, Carstairs decile
Rusconi [51]	2017	mutiple countries	Europe	NA	NA	5–9	Sex, GA, weight for GA, birth year, birth country	Age, parity, education, pregnancy (smoking, BMI before, and diabetes), HDP, asthma
Lavin [20]	2017	India	Asia	289	1,717	8	Sex, term low BW	Age, liveborn parity, household smoking, cooking fuel, geographic location, livestock ownership, housing quality, household size, wealth index
		Vietnam		178	1,760			
Chen [50]	2017	China	Asia	6,556	13,143	5.5	Sex, GA, birth order	Age, education level, family monthly income
Peters [52]	2018	Australia	Oceania	107,560	185,883	5	Sex, GA (small or large), BW, birth trauma	Age, parity, pharmacological pain medication or anesthesia at birth, birth country, socioeconomic status
Liao [53]	2020	Australia	Oceania	2,138	4,651	6–7	GA, BW	Age, pregnancy smoking, birth country, Socio-economic Indexes for Areas
Soullane [55]	2021	Canada	North America	216,547	645,427	≤ 13	Sex	Age, parity, atopy, HDP, diabetes (pregnancy or preexisting), illicit use (drug, alcohol, and tobacco), socioeconomic disadvantage, time period
Brew [54]	2021	Australia	Oceania	5,755	20,018	1–4	NA	Asthma, medical conditions, remoteness
Salem [56]	2022	Switzerland	Europe	65	306	6	Sex, GA, BW, exclusive breastfeeding duration, older siblings, childcare attendance	Age, pregnancy smoking, atopy status, current smoking (parents)
Wang [19]	2023	China	Asia	305,890	583,303	9	GA, BW, birth length	Age, parity, allergic diseases, pregnancy diabetes, preeclampsia, urbanization levels
O’Connor [57]	2023	UK	Europe	3,873	14,340	7, 11, 14	GA (small)	Age, BMI, asthma, pregnancy smoking, HDP, diabetes, education, ethnicity, income quintile

*CS* cesarean section, *VD* vaginal delivery, *GA* gestational age, *BW* birth weight, *BMI* body mass index, *HDP* hypertensive disorders of pregnancy, *NA* not available

### Association of cesarean section and asthma

**Fig. 2.**
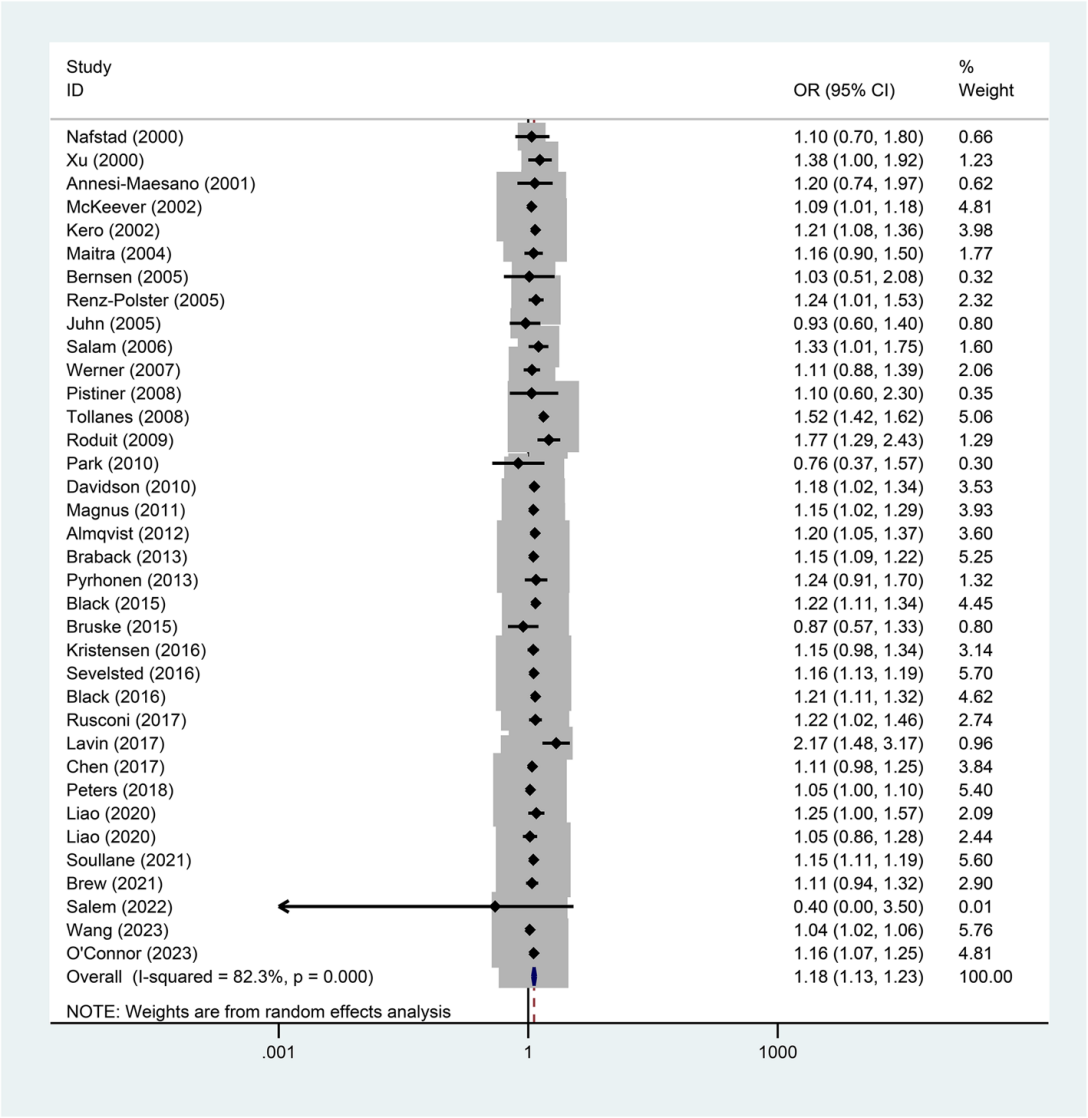
Forest plot of the association between cesarean section and asthma (*P* < 0.001)

Thirty-five articles reported on the association between mode of delivery and asthma. The results showed that the incidence of asthma was higher in offspring born via CS than those born via VD (OR = 1.18, 95%CI = 1.13–1.23, *P* < 0.001, I^2^ = 82.3%) (Fig. 2). To explore the sources of heterogeneity, the following subgroup analyses were designed (Table 2). First, subgroup analysis based on CS type showed that offspring born via elective CS (OR = 1.18, 95%CI = 1.11–1.25) and emergency CS (OR = 1.18, 95%CI = 1.10–1.27) had a higher incidence of asthma than the VD group, which was consistent with the overall results. According to sex-grouped data, female offspring born via CS (OR = 1.26, 95%CI = 1.13–1.42) had a higher incidence of asthma compared to the VD group, but there was no difference in males (OR = 1.07, 95%CI = 0.94–1.22). According to the continental divisions in the different study regions, there was no difference in asthma incidence between Asian populations born via CS (OR = 1.17, 95%CI = 0.97–1.42) and offspring born via VD. Those born via CS in Europe, North America and Oceania all had a higher incidence of asthma than the VD group (*P* < 0.05).

**Table 2:** Subgroup analyses of the association between cesarean section and asthma

	No. of studies	OR	95%CI	*p*	Heterogeneity (I^2^) (%)
Type of CS					
Elective CS	13	1.18	1.11–1.25	< 0.001	71.4
Emergency CS	13	1.18	1.10–1.27	< 0.001	83.3
Sex					
Female	3	1.26	1.13–1.42	< 0.001	0
Male	3	1.07	0.94–1.22	0.325	0
Continent					
Asia	4	1.17	0.97–1.42	0.102	81.3
Europe	23	1.20	1.15–1.26	< 0.001	70.4
North America	5	1.15	1.11–1.19	< 0.001	0
Oceania	3	1.06	1.02–1.11	0.008	0
Atopic asthma	6	1.14	1.11–1.18	< 0.001	0
Persistent asthma	3	1.15	0.99–1.33	0.063	65.3
Cohort design					
Prospective cohort	13	1.19	1.11–1.28	< 0.001	40.4
Retrospective cohort	22	1.17	1.11–1.23	< 0.001	86.9

*OR* odds ratio, *CI* confidence interval, *CS* cesarean section

Offspring born via CS had a higher incidence of atopic asthma (OR = 1.14, 95%CI = 1.11–1.18) compared to the VD group. The CS group had a higher incidence of persistent asthma (OR = 1.15, *P* = 0.063) than the VD group, but this difference was not statistically significant. Finally, in prospective cohort studies (OR = 1.19, 95%CI = 1.11–1.28) and retrospective cohort studies (OR = 1.17, 95%CI = 1.11–1.23), the incidence of asthma in offspring born via CS was higher than that in controls.

### Publication bias and sensitivity analyses

The statistical results of the Begg’s funnel plot showed no potential publication bias in the forest plot of the relationship between mode of delivery and incidence of asthma (*P* = 0.066) (SFig. 1). After excluding articles one by one, sensitivity analysis showed stable results (SFig. 2).

## Discussion

The purpose of this study was to investigate the statistical association between CS and asthma in children/adolescents, which was used to infer whether there is an effect of CS on asthma. The results of the systematic review and meta-analysis showed that children/adolescents born via CS were at increased incidence of developing asthma compared to VD. Further subgroup analyses showed that the relationship between CS and asthma was not affected by CS type, asthma type, or cohort design. But the increased incidence of asthma in children/adolescents born via CS may be influenced by sex and region.

The etiology of asthma has not been determined, and some studies believe that both genetic factors and environmental factors affect the occurrence of asthma [6]. Most immune system dysplasia is caused by environmental factors [58], which is an important cause of the epidemic of noncommunicable diseases [59]. Herein, the possible mechanisms by which CS increases asthma incidence are speculated from the following aspects.

First, childbirth is one of the early exposures for newborns. As is known to all, newborns born via CS are exposed to a different external environment for the first time compared to VD. Neonates born via VD are primarily exposed to bacteria in and around the maternal birth canal, whereas neonates born via CS are predominantly exposed to external bacteria [60]. Animal studies have shown that CS affects the diversity and density of the intestinal flora [61]. Infants born with CS have reduced numbers of Bacteroides and microbial sphingolipids in their faeces, so infants are more susceptible to asthma [62].

Second, compared to VD, CS was considered to postpone the onset of breastfeeding and to shorten the duration of exclusive breastfeeding [63], which may result in infants having insufficient exposure to breast milk. Breast milk contains high amounts of immunoglobulin (Ig) A, glycans [64], bioactive enzymes, and hormones that benefit the development of the immature immune defense system [65]. Breast milk has been found to transfer airborne antigens to newborn mice. Due to the presence of transforming growth factor-β mediated by CD4 + T lymphocytes in breast milk, its signalling is dependent on T cells. This induces antigen tolerance in newborns and provides specific protection against some allergic airway diseases, such as asthma [66]. Moreover, adequate breastfeeding is thought to facilitate the growth of infants’ lungs [67]. Therefore, insufficient breastfeeding may increase the risk of asthma.

Third, CS increases the binding of the progeny dopamine D1-like-receptor [68]. The conduction signal of D1-like-receptor facilitate the activation of the B-cell activating transcription factor, thereby increasing the transcription of the retinoic acid receptor-related orphan receptor-γ-t, and promoting the differentiation of T helper cell (Th) 17. Correspondingly, more Th17 were found in the spleen cells of mice in the asthma group than in the control group [69]. Th17 participate in antigen-induced aggregation of neutrophils and eosinophils in the airways, which play an important role in asthma [70, 71]. Antagonizing D1-like-receptor will inhibit the Th17-mediated inflammatory response in the lungs [72], but this evidence has not yet been validated in humans.

Finally, infants born via CS exhibit higher DNA methylation of cord blood leukocytes [73]. DNA methylation has been shown to play an important role in fetal development and may be an important cause of susceptibility to certain diseases [74]. DNA methylation may alter the composition of immune cells by regulating gene expression, putting CS offspring at higher risk of asthma. Undesirable methylation may disrupt the balance of Th1 and Th2, thereby increasing the risk of immune disease, which may be a regulatory mechanism for allergic asthma [75]. Allergic asthma is also known as atopic asthma, and the subgroup results in this study show that CS may be a risk factor for atopic asthma.

Subgroup results showed that the incidence of asthma appears to vary by sex. Compared to VD, CS is a risk factor for asthma in female rather than in male. There may be three possible reasons for this. (1) Asthma is a heterogeneous condition that may be sex-specific. Biological differences in development in the womb may explain the sex differences in asthma incidence. Sex influences the physiology and development of the infant’s lungs [76, 77]. In addition, females will appear more asthma attacks and asthma symptoms compared to males, and bronchial hyperresponsiveness is more common in females [78]. Asthma attacks in female appear to be closely related to menstrual periods. Asthma symptoms worsen during ovulation and menstruation [79]. Asthma patients have high markers of inflammation during the menstrual cycle, so asthma may be associated with female physiological hormones [80]. (2) Female infants have better viability than male infants when faced with adverse birth circumstances such as prematurity [81], which may result in more female babies surviving than male infants. Thus, more female than male infants are registered as having asthma. This may be one of the reasons why no association was found between CS male offspring and asthma. (3) The number of relevant studies that could be included was small and the results were subject to some chance.

The prevalence of childhood asthma varies considerably between countries [82]. It is well known that the developed countries are mainly distributed in Europe. Of the studies included in this systematic review and meta-analysis, the largest number of studies, up to twenty-three, were conducted in Europe. Notably, these studies were carried out in developed countries. In addition, research in North America and Oceania was also carried out in developed countries. Our results show that infants born via CS have a higher incidence of asthma compared to VD births in Europe, North America, and Oceania. Interestingly, no such association was found in infants born in Asia. The following three reasons are considered: (1) race may have influenced the onset of asthma. The relative prevalence of asthma varies by ethnic group [83]. (2) Developed countries have a high level of medical care and pay more attention to the health damage caused by diseases. People actively seek medical treatment, which is conducive to the diagnosis of diseases. This may be one of the reasons for the largest increase in asthma incidence in developed countries [84]. Developing country studies make up the majority of Asian regional studies. Disease diagnosis rates in developing countries may be lower due to a variety of factors. (3) And it should also be considered that the criteria for performing CS may vary between developing and developed countries.

Reviewing past systematic review and meta-analyses, Keag et al.concluded that CS was associated with asthma in children under 12 years of age. However, the number of studies included in this meta-analysis is relatively small [12]. A subsequent study focused on the relationship between CS and asthma. In addition to cohort studies, this meta-analysis included case–control studies and cross-sectional studies [85], which may have reduced the level of evidence for the results. Another meta-analysis on a European population took into account the heterogeneity of results and concluded that CS could not be clearly recognized as a risk factor for asthma in children [22].

The strengths of this study are as follows: (1) the number of included studies was comprehensive. It was an update and supplement to previous meta-analyses with detailed subgroup analyses. (2) This article was a systematic review and meta-analysis based on cohort studies. The high certainty of the evidence from the cohort studies contributed to the credibility of this study. However, there are some limitations: (1) there was selection bias and follow-up bias in the original studies. (2) The number of relevant studies in some subgroups was small.

Conclusions: CS seems to be associated with asthma in children/adolescent offspring compared to VD. However, the result has a relatively high degree of heterogeneity and require further validation. Subgroup analyses showed that sex may influence the relationship between CS and asthma, with the risk of asthma in CS offspring only present in females. The risk of CS for asthma appears to differ across regions. CS may be related to childhood/adolescent asthma in populations in Europe, North America, and Oceania.

### Supplementary Information

**Additional file 1:** **Supplementary Table 1. **Search Strategy.

**Additional file 2:** **Supplementary Table 2.**Characteristics of all the studies included in the systematic review and meta-analysis.

**Additional file 3:** **Supplementary Table 3.** Quality assessment of cohort studies included.

**Additional file 4:** **SFigure 1.** Publication bias of the association between cesarean section and asthma (*P*=0.066).

**Additional file 5:** **SFigure 2.** Sensitivity analysis of the association between cesarean section and asthma. 

## Data Availability

The datasets supporting this article’s conclusions are included within the article and its additional files.

## Abbreviations

CS: Cesarean section
VD: Vaginal delivery
PRISMA: Preferred Reporting Items for Systematic Reviews and Meta-Analyses
NOS: Newcastle-Ottawa Quality Assessment Scale
OR: Odds ratio
CI: Confidence interval
Ig: Immunoglobulin
Th: T helper cell
GA: Gestational age
BW: Birth weight
BMI: Body mass index
HDP: Hypertensive disorders of pregnancy
LSAC: The Longitudinal Study of Australian Children
P: Prospective cohort
R: Retrospective cohort
ICD: The International Classification of Diseases
CM: Clinical Modification
AM: Australian Modification
ISAAC: The International Study of Asthma and Allergies in Childhood
ICS: Inhaled corticosteroid
NA: Not available
