# Supplementary material for: Association of cesarean section with asthma in children/adolescents: a systematic review and meta-analysis based on cohort studies
Source: BMC Pediatr. 2023 Nov 16;23:571. doi: 10.1186/s12887-023-04396-1 (PMC10652517; doi:10.1186/s12887-023-04396-1)
Supplement: Supplementary file 1 — Additional file 1: Supplementary Table 1. Search Strategy. [file 12887_2023_4396_MOESM1_ESM.docx]

**Supplementary Table 1. Search Strategy.**

**Data searched from PubMed**

| Search Query [All Fields] | Results |
| --- | --- |
| #1 Cesarean Section OR Cesarean Sections OR Delivery, Abdominal OR Abdominal Deliveries OR Deliveries, Abdominal OR Caesarean Section OR Caesarean Sections OR Abdominal Delivery OR C-Section OR C Section OR C-Sections OR Postcesarean Section OR cesarean delivery OR mode of delivery OR perinatal | 11,694 |
| #2 Asthma OR Asthmas OR Bronchial Asthma OR Asthma, Bronchial | 15,483 |
| #3 #1 AND #2 | 1,507 |

Searched on February 1, 2023. Results: 1,507.

**Data searched from Web of Science**

| Search Query [Topic] | Results |
| --- | --- |
| #1 Cesarean Section OR Cesarean Sections OR Delivery, Abdominal OR Abdominal Deliveries OR Deliveries, Abdominal OR Caesarean Section OR Caesarean Sections OR Abdominal Delivery OR C-Section OR C Section OR C-Sections OR Postcesarean Section OR cesarean delivery OR mode of delivery OR perinatal | 17,485 |
| #2 Asthma OR Asthmas OR Bronchial Asthma OR Asthma, Bronchial | 19,257 |
| #3 #1 AND #2 | 2,019 |

Searched on February 1, 2023. Results: 2,019.

**Data searched from** **Embase**

| Search Query [exp] | Results |
| --- | --- |
| #1 Cesarean Section OR Cesarean Sections OR Delivery, Abdominal OR Abdominal Deliveries OR Deliveries, Abdominal OR Caesarean Section OR Caesarean Sections OR Abdominal Delivery OR C-Section OR C Section OR C-Sections OR Postcesarean Section OR cesarean delivery OR mode of delivery OR perinatal | 12,574 |
| #2 Asthma OR Asthmas OR Bronchial Asthma OR Asthma, Bronchial | 16,482 |
| #3 #1 AND #2 | 1,529 |

Searched on February 1, 2023. Results: 1,529.

**Data searched from Cochrane Library**

| Search Query [ti, ab, kw] | Results |
| --- | --- |
| #1 Cesarean Section OR Cesarean Sections OR Delivery, Abdominal OR Abdominal Deliveries OR Deliveries, Abdominal OR Caesarean Section OR Caesarean Sections OR Abdominal Delivery OR C-Section OR C Section OR C-Sections OR Postcesarean Section OR cesarean delivery OR mode of delivery OR perinatal | 9,854 |
| #2 Asthma OR Asthmas OR Bronchial Asthma OR Asthma, Bronchial | 10,482 |
| #3 #1 AND #2 | 1,073 |

Searched on February 1, 2023. Results: 1,073.
