# Supplementary material for: Association of cesarean section with asthma in children/adolescents: a systematic review and meta-analysis based on cohort studies
Source: BMC Pediatr. 2023 Nov 16;23:571. doi: 10.1186/s12887-023-04396-1 (PMC10652517; doi:10.1186/s12887-023-04396-1)
Supplement: Supplementary file 2 — Additional file 2: Supplementary Table 2.Characteristics of all the studies included in the systematic review and meta-analysis. [file 12887_2023_4396_MOESM2_ESM.docx]

**Supplementary Table 2. Characteristics of all the studies included in the systematic review and meta-analysis.**

| Author | Year | Cohort design | Data source | Birth period | Follow-up time (years) | Registration of asthma |
| --- | --- | --- | --- | --- | --- | --- |
| Nafstad[25] | 2000 | P | The Oslo Birth Cohort | 1992-1993 | 4 | Questionnaire (physician’s diagnosis; symptoms) |
| Xu[26] | 2000 | P | A population-based prospective birth cohort in northern Finland | 1985.7-1986.6 | 7 | Parental questionnaire; Hospital records |
| Annesi-Maesano[27] | 2001 | R | The National Child Development Study | 1958.3 | NA | Standardised questionnaire (ISAAC) |
| McKeever  [29] | 2002 | R | The West Midlands General Practice Research Database | NA | 2.9 (0-11) | ICD-8 and Read codes (hierarchical codes) |
| Kero[28] | 2002 | R | Finnish 1987 Medical Birth Register | 1987–1995 | 7 | Hospital Discharge Register, Statistical Database for Care Support and from Statistical Database for Preferential Refunded Medicines (ICD-9 code 493; ICD-9 code 493) |
| Maitra[30] | 2004 | P | The Avon Longitudinal Study of Parents and Children | 1991.4.1-1992.12.31 | 8 | Questionnaire (physician’s diagnosis) |
| Bernsen[31] | 2005 | R | The files of a Municipal Health Service in the Netherlands | 1988-1990 | 6 | Parental questionnaire/physician's report |
| Renz-Polster[33] | 2005 | R | The electronic medical records of a large health maintenance organization, Kaiser Permanente Northwest Region | 1990.1.1-1992.12.31 | 3-10 | Electronic medical records |
| Juhn[32] | 2005 | R | The learning disability study children who were residents of the City of Rochester | 1976.1.1-1982.12.31 | 7 | Physician’s diagnosis; symptoms; test |
| Salam[34] | 2006 | R | Children born in California and who participated in the Children’s Health Study | 1975-1987 | ＞8 | Parental report (physician’s diagnosis) |
| Werner[35] | 2007 | P | The ‘Healthy Habits for Two’ cohort | 1984-1987 | 15-18 | Questionnaire |
| Pistiner[36] | 2008 | P | A metropolitan Boston prospective birth cohort study | 1994.9-1996.8 | 9 | Questionnaire (physician’s diagnosis) |
| Tollånes[37] | 2008 | R | The Medical Birth Registry of Norway | 1967-1998 | 18 | ICD9 code 493; ICD10 code J45 |
| Roduit[38] | 2009 | P | The Prevention and Incidence of Asthma and Mite Allergy study | 1996.5-1997.12 | 8 | Standardised questionnaire (symptoms; therapy; physician’s diagnosis) |
| Park[40] | 2010 | R | Children attending Severance Children’s Hospital, Yonsei University College of Medicine | NA | NA | Physician’s diagnosis; symptoms |
| Davidson[39] | 2010 | R | The Oxford record linkage study | 1970–1989 | ＞10 | Hospital admission records (ICD-8,9 codes 493; ICD-10 J45 and J46) |
| Magnus[41] | 2011 | P | The Norwegian Mother and Child Cohort | 2001-2007 | NA | Parental questionnaire |
| Almqvist[42] | 2012 | R | The Swedish national registers held by the Swedish National Board of Health and Welfare and Statistics Sweden | 1993.6-1999.6 | ＞10 | ICD-9 codes 493; ICD-10 J45-J46; any asthma medication (code R03) |
| Bråbäck[43] | 2013 | R | The Swedish Medical Birth Register | 1999.1.1–2006.12.31 | 2-10 | Swedish Prescribed Drug Register (ICS) |
| Pyrhönen[44] | 2013 | R | The South Karelian Allergy Research Project | 2001.4-2005.3 | ＜5 | Questionnaire (symptoms/physician’s diagnosis); allergy test results |
| Black[45] | 2015 | R | The Scottish Morbidity Record database | 1993.1.1–2007.12.31 | 14.8 | Prescriptions (salbutamol inhalers) |
| Brüske[46] | 2015 | P | The German Infant Nutrition Intervention Study Plus | 1995–1998 | 15 | Questionnaire (physician’s diagnosis/medication); spirometry tests |
| Kristensen  [48] | 2016 | P | The Danish National Birth Registry | 1997.1-2012.12 | 14 | ICD-10 codes J45.0-J45.9 |
| Sevelsted[49] | 2016 | P | The Copenhagen Prospective Studies on Asthma in Childhood2000 | 1997-2010 | 15 | ICS medication (code R03); outpatient or in-hospital diagnoses; physician’s diagnosis |
| Black[47] | 2016 | R | The Scottish Morbidity Record 02 | 1993.1.1-2007.12.31 | 21 | Discharge diagnosis |
| Rusconi[51] | 2017 | R | Nine European birth cohorts | 1996-2006 | 5-9 | Parental questionnaire (ISAAC); symptoms |
| Lavin[20] | 2017 | P | Multi-national longitudinal cohort study (the Young Lives Study) in two low-to-middle-income countries | 2001-2002 | 8 | Outpatient or in-hospital diagnoses |
| Chen[50] | 2017 | R | The Taiwan Birth Cohort Study | 2005 | 5.5 | Physician’s diagnosis |
| Peters[52] | 2018 | R | Children born in New South Wales, Australia | 2000.1.1-2008.8.31 | 5 | ICD-10-AM |
| Liao[53] | 2020 | P | The HealthNuts study and LSAC | HealthNuts: 2006-2010  LSAC: 2003-2004 | ＞6 | Diagnoses of hospital |
| Soullane[55] | 2021 | R | Singleton children born at term in the hospitals of Quebec, Canada | 2006-2019 | 13 | In-hospital diagnoses |
| Brew[54] | 2021 | R | Australian Aboriginal children | 2003-2012 | ＜5 | ICD-10- AM code J45 or J46; symptom |
| Salem[56] | 2022 | P | The Bern-Basel Infant Lung Development birth cohort | 1999.4-2019.5 | 6 | Questionnaire (ISAAC) to Clinician interview |
| Wang[58] | 2023 | R | Taiwan Birth Cohort Study database and the National Health Insurance Research Database | 2006-2009 | ＞6 | Outpatient or in-hospital diagnoses (ICD-9-CM code 493.X; ICD-10-CM code J45) |
| O'Connor[57] | 2023 | R | Millennium Cohort Study | 2000-2002 | NA | Questionnaire (ISAAC) |

LSAC, the Longitudinal Study of Australian Children; P, prospective cohort; R, retrospective cohort; ICD, the International Classification of Diseases; CM, Clinical Modification; AM, Australian Modification; ISAAC, the International Study of Asthma and Allergies in Childhood; ICS, inhaled corticosteroid; NA, not available.
