# Supplementary material for: Association of cesarean section with asthma in children/adolescents: a systematic review and meta-analysis based on cohort studies
Source: BMC Pediatr. 2023 Nov 16;23:571. doi: 10.1186/s12887-023-04396-1 (PMC10652517; doi:10.1186/s12887-023-04396-1)
Supplement: Supplementary file 3 — Additional file 3: Supplementary Table 3. Quality assessment of cohort studies included. [file 12887_2023_4396_MOESM3_ESM.docx]

**Supplementary Table 3.** **Quality assessment of cohort studies included.**

| Author, year,  Study (Cohort study) | **Selection (Out of 4)** | | | | **Comparability**  **(Out of 2)** | **Outcomes (Out of 3)** | | | **Total**  **(Out of 9)** |
| --- | --- | --- | --- | --- | --- | --- | --- | --- | --- |
|  | Representativeness of exposed cohort | Selection of non exposed cohort | Ascertainment  of exposure | Outcome not present at the start of the study |  | Assessment of outcomes | Length of follow-up | Adequacy of follow up of cohorts |  |
| Nafstad 2000[25] | 1 | 1 | 1 | 1 | 1 | 1 | 1 | 0 | 7 |
| Xu 2000[26] | 1 | 1 | 1 | 1 | 2 | 1 | 1 | 1 | 9 |
| Annesi-Maesano 2001  [27] | 1 | 1 | 0 | 1 | 1 | 1 | 1 | 1 | 7 |
| McKeever 2002[29] | 1 | 1 | 1 | 1 | 0 | 1 | 1 | 0 | 6 |
| Kero 2002[28] | 1 | 1 | 1 | 1 | 1 | 1 | 1 | 1 | 8 |
| Maitra 2004[30] | 1 | 1 | 1 | 1 | 2 | 1 | 1 | 1 | 9 |
| Bernsen 2005  2  2005  [31] | 1 | 1 | 1 | 1 | 2 | 1 | 1 | 1 | 9 |
| Renz-Polster 2005[33] | 1 | 1 | 1 | 1 | 2 | 1 | 1 | 1 | 9 |
| Juhn 2005[32] | 1 | 1 | 1 | 1 | 1 | 1 | 1 | 0 | 7 |
| Salam 2006[34] | 1 | 1 | 1 | 1 | 2 | 1 | 1 | 0 | 8 |
| Werner 2007[35] | 1 | 1 | 1 | 1 | 2 | 1 | 1 | 1 | 9 |
| Pistiner 2008[36] | 1 | 1 | 1 | 1 | 0 | 1 | 1 | 1 | 7 |
| Tollånes 2008[37] | 1 | 1 | 1 | 1 | 1 | 1 | 1 | 1 | 8 |
| Roduit 2009[38] | 1 | 1 | 0 | 1 | 1 | 1 | 1 | 0 | 6 |
| Park 2010[40] | 1 | 1 | 1 | 1 | 2 | 1 | 1 | 0 | 8 |
| Davidson 2010  201  [39] | 1 | 1 | 1 | 1 | 2 | 1 | 1 | 0 | 8 |
| Magnus 2011[41] | 1 | 1 | 1 | 1 | 2 | 1 | 0 | 0 | 7 |
| Almqvist 2012[42] | 1 | 1 | 1 | 1 | 2 | 1 | 1 | 1 | 9 |
| Bråbäck 2013[43] | 1 | 1 | 1 | 1 | 2 | 1 | 0 | 0 | 7 |
| Pyrhönen 2013[44] | 1 | 1 | 1 | 1 | 1 | 1 | 0 | 0 | 6 |
| Black 2015[45] | 1 | 1 | 1 | 1 | 1 | 1 | 1 | 1 | 8 |
| Brüske 2015[46] | 1 | 1 | 1 | 1 | 1 | 1 | 1 | 0 | 7 |
| Kristensen 2016[48] | 1 | 1 | 1 | 1 | 1 | 1 | 1 | 1 | 8 |
| Sevelsted 2016[49] | 1 | 1 | 1 | 1 | 1 | 1 | 1 | 0 | 7 |
| Black 2016[47] | 1 | 1 | 1 | 1 | 1 | 1 | 1 | 1 | 8 |
| Rusconi 2017[51] | 1 | 1 | 1 | 1 | 2 | 1 | 1 | 0 | 8 |
| Lavin 2017[20] | 1 | 1 | 1 | 1 | 2 | 1 | 1 | 1 | 9 |
| Chen 2017[50] | 1 | 1 | 1 | 1 | 1 | 1 | 0 | 0 | 6 |
| Peters 2018[52] | 1 | 1 | 1 | 1 | 2 | 1 | 0 | 0 | 7 |
| Liao 2020[53] | 1 | 1 | 0 | 1 | 2 | 1 | 1 | 1 | 8 |
| Soullane 2021[55] | 1 | 1 | 1 | 1 | 2 | 1 | 1 | 1 | 9 |
| Brew 2021[54] | 1 | 1 | 1 | 1 | 2 | 1 | 1 | 1 | 9 |
| Salem 2022[56] | 1 | 1 | 1 | 1 | 2 | 1 | 1 | 1 | 9 |
| Wang 2023[58] | 1 | 1 | 1 | 1 | 2 | 1 | 1 | 1 | 9 |
| O'Connor 2023[57] | 1 | 1 | 0 | 1 | 2 | 1 | 1 | 0 | 7 |

The observational studies were assessed by the Newcastle-Ottawa Quality Assessment Scale (NOS) checklist of cohort studies.
