# Supplementary figures and images for: Association of cesarean section with asthma in children/adolescents: a systematic review and meta-analysis based on cohort studies
Source: BMC Pediatr. 2023 Nov 16;23:571. doi: 10.1186/s12887-023-04396-1 (PMC10652517; doi:10.1186/s12887-023-04396-1)

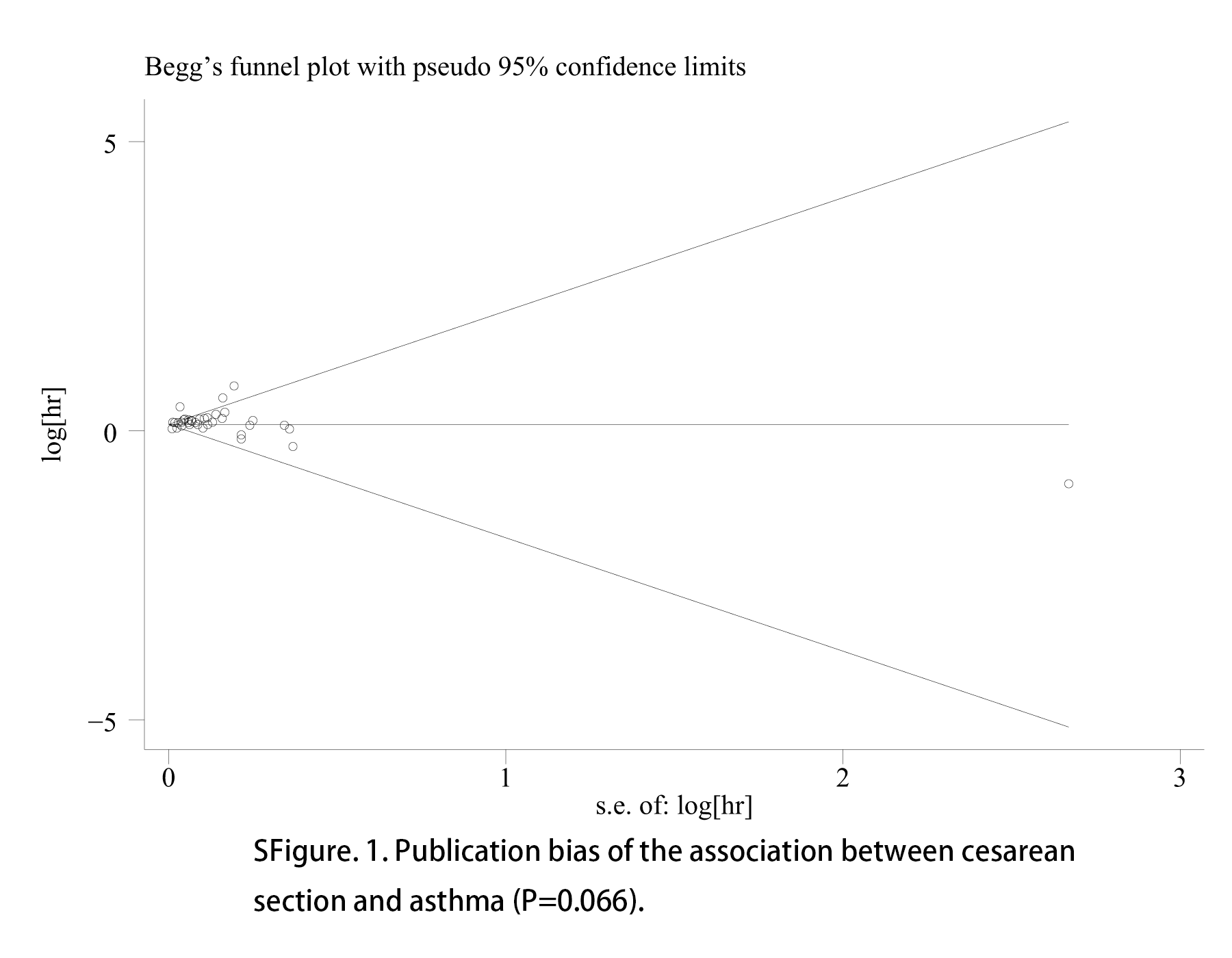

Supplement: Supplementary file 4 — Additional file 4: SFigure 1. Publication bias of the association between cesarean section and asthma (P=0.066). [file 12887_2023_4396_MOESM4_ESM.tif]

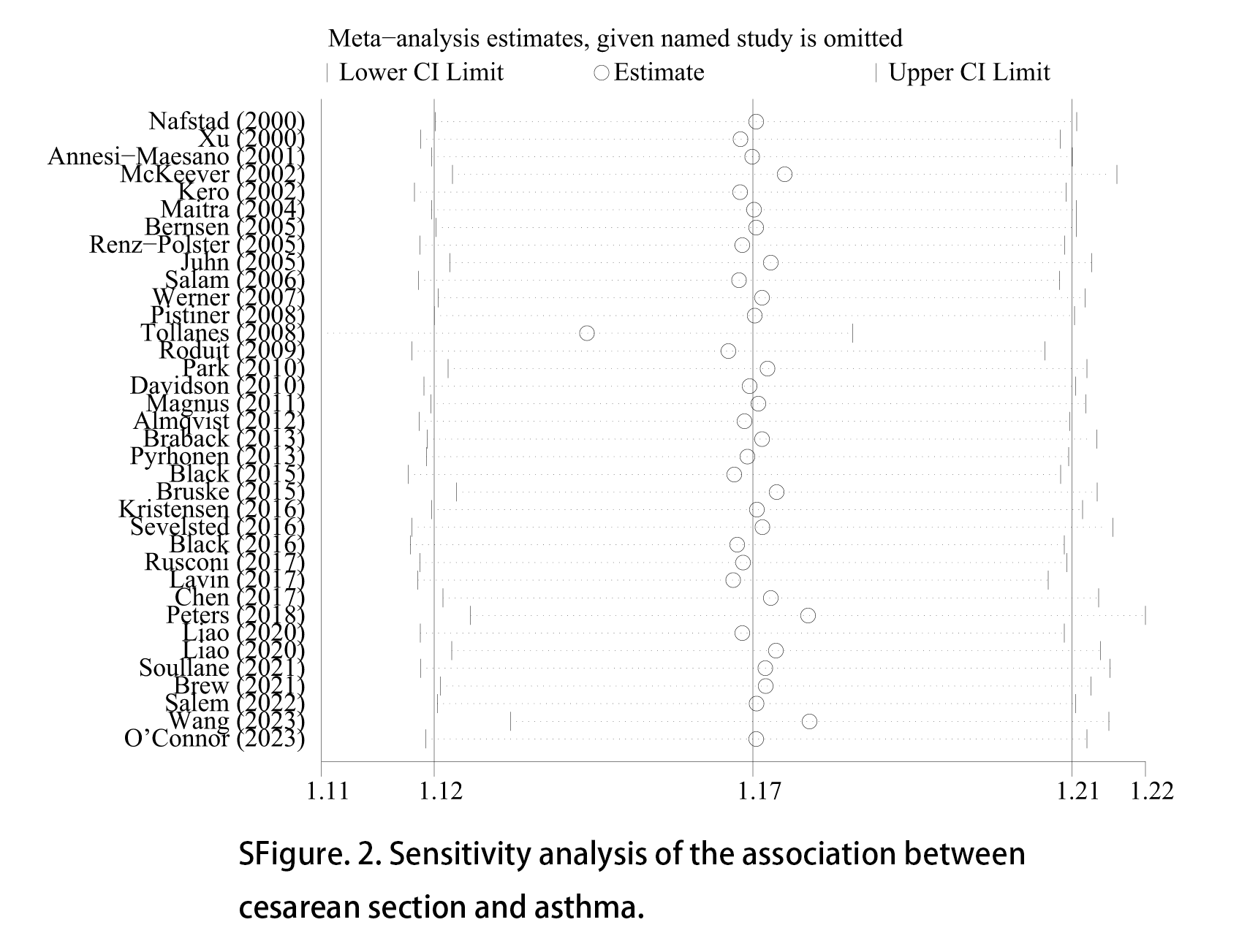

Supplement: Supplementary file 5 — Additional file 5: SFigure 2. Sensitivity analysis of the association between cesarean section and asthma. [file 12887_2023_4396_MOESM5_ESM.tif]
